# Pyrroline-5-Carboxylate Reductase 1 Directs the Cartilage Protective and Regenerative Potential of Murphy Roths Large Mouse Mesenchymal Stem Cells

**DOI:** 10.3389/fcell.2021.604756

**Published:** 2021-07-02

**Authors:** Gautier Tejedor, Rafael Contreras-Lopez, Audrey Barthelaix, Maxime Ruiz, Danièle Noël, Frédéric De Ceuninck, Philippe Pastoureau, Patricia Luz-Crawford, Christian Jorgensen, Farida Djouad

**Affiliations:** ^1^IRMB, INSERM, University Montpellier, Montpellier, France; ^2^CHU Montpellier, Montpellier, France; ^3^Center for Therapeutic Innovation, Immuno-Inflammatory Disease, Institut de Recherches Servier, Croissy-sur-Seine, France; ^4^Laboratorio de Inmunología Celular y Molecular, Facultad de Medicina, Universidad de los Andes, Santiago, Chile

**Keywords:** MRL mouse, regeneration, mesenchymal stem cells, PYCR1, metabolism, chondrogenesis, chondrocyte, chondroprotection

## Abstract

Murphy Roths Large (MRL) mice possess outstanding capacity to regenerate several tissues. In the present study, we investigated whether this regenerative potential could be associated with the intrinsic particularities possessed by their mesenchymal stem cells (MSCs). We demonstrated that MSCs derived from MRL mice (MRL MSCs) display a superior chondrogenic potential than do C57BL/6 MSC (BL6 MSCs). This higher chondrogenic potential of MRL MSCs was associated with a higher expression level of pyrroline-5-carboxylate reductase 1 (PYCR1), an enzyme that catalyzes the biosynthesis of proline, in MRL MSCs compared with BL6 MSCs. The knockdown of PYCR1 in MRL MSCs, using a specific small interfering RNA (siRNA), abolishes their chondrogenic potential. Moreover, we showed that PYCR1 silencing in MRL MSCs induced a metabolic switch from glycolysis to oxidative phosphorylation. In two *in vitro* chondrocyte models that reproduce the main features of osteoarthritis (OA) chondrocytes including a downregulation of chondrocyte markers, a significant decrease of PYCR1 was observed. A downregulation of chondrocyte markers was also observed by silencing PYCR1 in freshly isolated healthy chondrocytes. Regarding MSC chondroprotective properties on chondrocytes with OA features, we showed that MSCs silenced for PYCR1 failed to protect chondrocytes from a reduced expression of anabolic markers, while MSCs overexpressing PYCR1 exhibited an increased chondroprotective potential. Finally, using the ear punch model, we demonstrated that MRL MSCs induced a regenerative response in non-regenerating BL6 mice, while BL6 and MRL MSCs deficient for PYCR1 did not. In conclusion, our results provide evidence that MRL mouse regenerative potential is, in part, attributed to its MSCs that exhibit higher PYCR1-dependent glycolytic potential, differentiation capacities, chondroprotective abilities, and regenerative potential than BL6 MSCs.

## Introduction

The superhealer Murphy Roths Large (MRL) mice possess remarkable capacity to regenerate several musculoskeletal tissues such as ear wounds, amputated digits, and injured articular cartilage with no evidence of scarring (Clark et al., 1998; Fitzgerald et al., 2008; Ward et al., 2008; Kwiatkowski et al., 2016; Deng et al., 2019; Sinha et al., 2019). Although the mechanisms that underlie MRL mice regenerative potential have been intensively studied during the two last decades, the exact process involved is still poorly understood.

Cartilage regeneration requires an extensive tissue remodeling; and in this context, the capacity of MRL mice to induce a breakdown in the basement membrane, which permits the formation of a blastema and ear-hole closure, has been shown (Gourevitch et al., 2003). This process relies on an inflammatory response characterized by the recruitment and the activation of neutrophils and macrophages positive for MMP-2, MMP-9, TIMP-2, and TIMP-3 in the ear after injury (Gourevitch et al., 2003). Thus, during the regenerative healing, an inflammatory regenerative environment with an increased number of pro-inflammatory cells in the MRL mice compared with the non-regenerating C57BL/6 mice was observed (Gourevitch et al., 2014). Moreover, the high regenerative potential of MRL mice has been attributed, in part, to their mesenchymal stem cells (MSCs) or their secretome (Diekman et al., 2013; Wang et al., 2020). The intra-articular injection of MSCs derived either from C57BL/6 mouse (B6 MSCs) or MRL mouse (MRL MSCs) prevents the development of post-traumatic arthritis after fracture at a similar extent, although MRL MSCs exhibit a higher capacity for bone volume increase during repair (Diekman et al., 2013).

Thus, MSCs and in particular MRL MSCs, described for their capacity to promote tissue repair/regeneration based on their trophic functions, should be further deciphered to identify promising therapeutic factors for degenerative diseases such as osteoarthritis (OA). Indeed, MSCs regulate the inflammatory response and provide a regenerative environment either by releasing bioactive molecules that will promote the functions of endogenous stem cells or by repressing the function and proliferation of abnormally activated immune cells (Djouad et al., 2009; Maumus et al., 2013a; Pers et al., 2015, 2018). MSC functions rely on their metabolic status. MSCs, when undifferentiated, rely on glycolysis for energy such as most types of stem cells. Then, MSCs activate the mitochondrial process of oxidative phosphorylation (OXPHOS) when induced to differentiate into osteoblasts (Shum et al., 2016). During the early phase of adipogenesis, MSCs exhibit an increased oxygen consumption and mitochondrial activity indicating a metabolic switch from glycolysis to OXPHOS (Drehmer et al., 2016). In contrast to MSCs induced to differentiate into osteoblasts or adipocytes, MSCs that differentiate into chondrocytes present reduced O_2_ consumption and OXPHOS, indicating an increased glycolysis (Pattappa et al., 2011). Therefore, given the pivotal role of MSC metabolic status on its functions, it is reasonable to hypothesize that the phenotypic and functional differences reported between MSCs from different sources (Elahi et al., 2016), species (Ren et al., 2009), or strain of mice (Peister et al., 2004; Bouffi et al., 2010) might be associated with metabolic differences. Moreover, the metabolic signature of MSCs is dynamic and changes with aging. Compared with rapid-aging MSCs, which display low regenerative capabilities, slow-aging MSCs exhibit a significantly higher glycolytic capacity and a higher potential for glucose uptake and reserve (Macrin et al., 2019).

In MRL mice, regeneration proceeds through the formation of a wound epithelium and a blastema-like structure, a heterogeneous cell mass that transiently forms adjacent to a specialized wound epithelium through migration and proliferation of local progenitor cells to give rise to distinct cell types organizing into the exact copy of the lost entity (for review, see Kumar and Brockes, 2012). Thus, regeneration such as tumorigenesis is characterized by a massive cell proliferation, tightly controlled in the case of regeneration and anarchic during tumorigenesis. Proline biosynthesis and metabolism play a central role in the metabolic reprogramming observed during tumorigenesis as well as during development (Phang and Liu, 2012; Phang et al., 2015; Phang, 2019). Pyrroline-5-carboxylate reductase 1 (PYCR1), a key enzyme for proline biosynthesis, is required for normal development. Indeed, PYCR1 mutation has been described in patients with multisystem disorders such as autosomal-recessive cutis laxa type 2 (ARCL2) characterized by premature aging, general developmental delay, and skin and joint laxity (Guernsey et al., 2009). Moreover, PYCR1 has been described to promote tumorigenesis. Indeed, PYCR1 inhibition using gene interference technology represses cell proliferation while promoting cell apoptosis in hepatocellular carcinoma (Zhuang et al., 2019). Similarly, PYCR1 silencing using small interfering RNA (siRNA) significantly inhibited cell proliferation and increased apoptosis of non-small cell lung cancer (Cai et al., 2018). PYCR1-dependent proline biosynthesis is pivotal for tumorigenesis by promoting cell proliferation and connecting the cycle of proline to glycolysis (Liu et al., 2015). However, the role of PYCR1 on MRL MSC regenerative process and metabolic status has never been investigated.

In this study, we addressed whether the regenerative potential of MRL mice could be attributed to the intrinsic properties of MSCs focusing on the role of PYCR1 in their therapeutic potential.

## Materials and Methods

### Bioethics

Mice were housed and cared for in accordance with the Ethics Committee on Animal Research and Care of the Languedoc-Roussillon. We obtained the approval from the Ethical Committee for animal experimentation of the Languedoc-Roussillon before initiating the study (approval CEEA-LR-12117).

### Mesenchymal Stem Cell Isolation and Expansion

Mesenchymal Stem Cells (MSCs) were isolated from MRL/Mpj and C57BL/6 mice bone marrow. Their expansion as well as their phenotypic and functional characterization was performed as previously described after their spontaneous immortalization *in vitro* (Bouffi et al., 2010; Tejedor et al., 2021). The immortalized MSCs used in the present study between passages 15 and 20 exhibited the minimal criteria for defining MSCs (Dominici et al., 2006).

### Chondrogenic Differentiation of Mesenchymal Stem Cell

Mesenchymal Stem Cell differentiation into chondrocytes was performed as previously described (Bouffi et al., 2010). Briefly, MSCs were induced to differentiate using the protocol of micropellets, which consist in seeding the MSCs at 2.5 × 10^5^ cells/well in 96-Well Polypropylene, centrifuged during 5 min at 400 *g*. The micropellets were cultured during 21 days in a medium containing DMEM (Invitrogen), 100 U/ml of penicillin/streptomycin, 10 μM of sodium-pyruvate, 1.7 μM of ascorbic acid-2-phosphate, insulin*-*transferrin*-*selenium (ITS; Sigma-Aldrich Corp., St. Louis, MO, United States), and 1 ng/ml of human Transforming Growth Factor β3 (hTGF-β3; R&D Systems, Minneapolis, MN, United States) prior to being recovered for RT-qPCR analysis.

### Chondrocyte Isolation and Expansion

Articular chondrocytes were isolated from femoral heads and knees of 3-day-old C57BL/6 mice and seeded at 25,000 cells/cm^2^ in 12-well TPP culture plates (TPP Techno Plastic Products, Trasadingen, Switzerland) in culture medium for 5 days as previously described (Gosset et al., 2008). The chondrocytes were treated with IL-1β (1 ng/ml, R&D Systems) during 24 h to reproduce the main features of OA chondrocytes. Then, IL-1β was removed, and IL-1β-induced chondrocytes were cocultured during another 24 h with MSCs seeded in culture inserts and recovered to be analyzed by RT-qPCR.

### Mesenchymal Stem Cell and Chondrocyte Transfection With Small Interfering RNA and Plasmids

Chondrocytes and MSCs were transfected at subconfluence (60%) with 200 nM of control siRNA (siCTL) or the siRNA against *Pycr1* (si*Pycr1*) (Silencer Select RNAi, Thermo Fisher Scientific, Illkirch, France) using oligofectamine reagent (Life Technologies, Courtaboeuf, France) according to the supplier’s recommendations.

Mesenchymal Stem Cells were transfected at 60% of confluence with control or PYCR1-expressing plasmids (pCMV6 Entry; OriGene Technologies, Rockville, MD, United States) using lipofectamine reagent (Life Technologies, Courtaboeuf) according to the supplier’s recommendations.

### Proliferation Assay

Murphy Roths Large MSC proliferation rate was assessed using the PrestoBlue assay (Promega, Charbonnières-les-Bains, France) and following the manufacturer’s recommendations. Briefly, MSCs were seeded at the density of 3,500 cells/cm^2^ in a 6-well plate 48 h after transfection, in a proliferative medium containing DMEM supplemented with 10% of fetal calf serum, 100 U/ml of penicillin/streptomycin, and 2 mmol/ml of glutamine. After 3 days of culture, MRL MSCs were collected, and the number of viable cells was quantified.

### RT-qPCR

Total RNA was isolated from each sample using RNeasy Mini Kit (Qiagen, Courtaboeuf, France), and the quantity and purity of the total RNA were determined by using a NanoDrop ND-1000 spectrophotometer (NanoDrop ND, Thermo Fisher Scientific). cDNA was synthesized by reverse transcribing 500 ng of RNA into cDNA using the SensiFAST cDNA synthesis kit (Bioline, Memphis, TN, United States). Quantitative PCR was performed using the SensiFAST^TM^ SYBR (Bioline) and a LightCycler^®^ 480 Detection system, following the manufacturer’s recommendations. Specific primers for *Acan*, *Adamts5*, *Col2B*, *Mmp13*, and *Pycr1* were designed using the Primer3 software (*Acan* F: GCGAGTCCAACTCTTCAAGC-R: GAAGTAGCAGGGGATGGTGA; *Adamts5* F: CTGCC TTCAAGGCAAATGTGTGG-R: CAATGGCGGTAGGCAAAC TGC; *Col2B* F: CTGGTGCTGCTGACGCT-R: GCCCT AATTTTCGGGCAT; *Mmp13* F: TCTGGATCACTCCAAGGAC C-R: ATCAGGAAGCATGAAATGGC; *Pycr1* F: GAAGAT GGCAGGCTTGTGGA-R: CTGGGAAGCCCCATTTTCAC. Data were normalized to the housekeeping gene ribosomal protein S9 (RPS9). Values were expressed as relative mRNA level of specific gene expression as obtained using the 2^–ΔCt^ method.

### Oxygen Consumption Rate and Extracellular Acidification Rate Measurement

Oxygen consumption rate (OCR) and extracellular acidification rate (ECAR) were measured using the XFe96 analyzer (Seahorse Bioscience, North Billerica, MA, United States). Murine MSCs (20,000 cells/well) were plated on 96-well plates, in XF media (non-buffered DMEM medium, without glucose, 2 mM of L-glutamine, and 1 mM of sodium pyruvate) and analyzed according to the manufacturer’s recommended protocol. Three independent readings were taken after each sequential injection. Instrumental background was measured in separate control wells using the same conditions without biologic material. ECAR/OCR ratio was calculated with the glycolytic rate and basal OCR.

### Lactate Quantification

Lactate was measured in the supernatants of MSCs using the Lactate assay kit II (Sigma Aldrich) following manufacturer’s instruction.

### Ear Punch Model

C57BL/6 female mice with an age of 10 weeks were used for the model. At day 0, we performed a reproducible ear hole with a 2-mm punch through the center of the ear. For the different groups, we injected either 20 μl of phosphate-buffered saline (PBS) (untreated) or 3 × 10^5^ MSCs/20 μl of PBS along the wound edge using a 10-μl Hamilton syringe connected with a 25-gauge needle (two injections were performed to inject a final volume of 20 μl). Measurements of the ear wound area were performed at day 0 and day 35 from using the ImageJ software on ear pictures. For the untreated condition, we injected PBS.

### Statistical Analysis

Generated *p*-values were obtained using the Mann–Whitney unpaired t-test, two-tailed, using GraphPad Prism 6 Software. Graphs show mean ± SEM. *p* < 0.05 (^∗^), *p* < 0.01 (^∗∗^), or *p* < 0.001 (^∗∗∗^) was considered statistically significant.

## Results

### Pycr1 Is Highly Expressed in Murphy Roths Large Mesenchymal Stem Cell and Progressively Increased During Chondrogenesis

**FIGURE 1:**
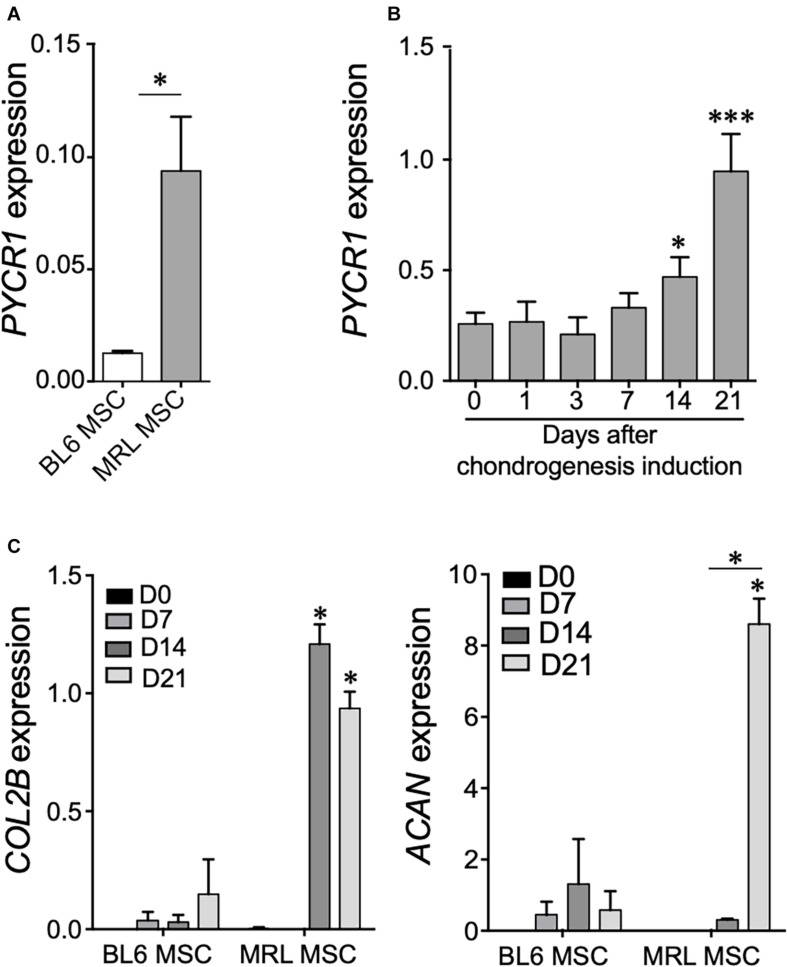
*Pycr1* is highly expressed in MRL MSCs and in MSC-derived chondrocytes. **(A)** *Pycr1* mRNA expression level in MRL MSCs and BL6 MSCs assessed by RT-qPCR. **(B)** *Pycr1* mRNA expression level in MRL MSCs at different time points of the chondrogenic differentiation of MRL MSCs induced in pellets by TGFβ3 assessed by RT-qPCR. **(C)** mRNA expression levels of chondrocyte markers, *Col2B* and *Acan*, in BL6 and MRL MSCs at different time points of the chondrogenic differentiation. Results represent the mean ± SEM of three independent experiments. Results represent the mean ± SEM of three independent experiments. Statistics: Mann–Whitney test, two-tailed. When not indicated day 0 (0) versus day 14 (14) or 21 (21), *p*-values < 0.05 (^∗^) or *p* < 0.001 (^∗∗∗^). MRL, Murphy Roths Large; MSCs, mesenchymal stem cells.

We first studied the expression level of *Pycr1* in adult MRL MSCs as compared with MSCs derived from BL6 mice by RT-qPCR. We found that *Pycr1* mRNA expression was significantly higher in MRL MSCs than in BL6 MSCs (Figure 1A). Then, since MSCs undergoing chondrogenesis rely on proline addition in the chondrogenic media and exhibit a metabolic shift toward glycolysis (Pattappa et al., 2011), we investigated whether *Pycr1* expression could be modulated during the induction of MSC differentiation into chondrocytes. *Pycr1* expression level was progressively increased during MSC chondrogenesis to reach a significantly higher level from D14 than undifferentiated MSCs (Figure 1B). This increased expression level of *Pycr1* paralleled the well-described increased expression level of the chondrocyte markers such as type IIB collagen (*Col2B*) and aggrecan (*Acan*) during MSC differentiation into chondrocytes (Figure 1C). Moreover, at day 21, we found that MRL MSCs induced to differentiate into chondrocytes express a higher level of *Col2B* and *Acan* than BL6 MSCs. This suggests that MRL MSCs exhibit a higher chondrogenic potential than BL6 MSCs (Figure 1C).

### Pycr1 Is Necessary for Mesenchymal Stem Cell Chondrogenic Potential

**FIGURE 2:**
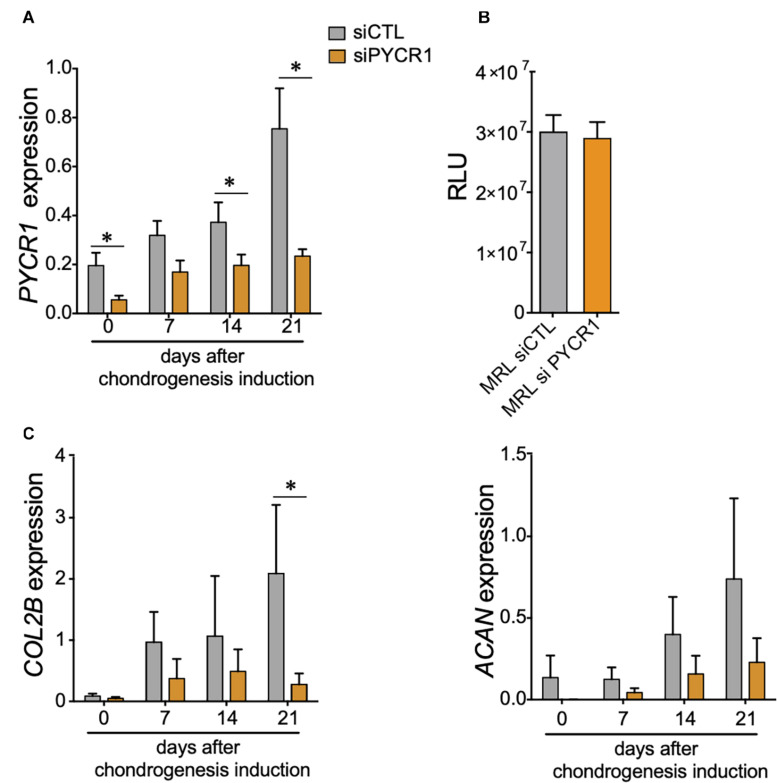
*Pycr1* is required for the chondrogenic potential of MRL MSCs. **(A)** *Pycr1* mRNA expression level in MRL MSCs transfected with either a control siRNA (siCTL) or a siRNA against *Pycr1* (siPYCR1) at different time points of the chondrogenic differentiation induced in pellets by TGFβ3. **(B)** Proliferation rate of MRL MSCs transfected with either a control siRNA (siCTL) or a siRNA against *Pycr1* (siPYCR1). **(C)** mRNA expression levels of chondrocyte markers, *Col2B* and *Acan*, in MRL MSCs transfected with either siCTL or siPYCR1 at different time points of the chondrogenic differentiation induced in pellets by TGFβ3. Results represent the mean ± SEM of three independent experiments. Statistics: Mann–Whitney test, two-tailed. *p*-Values < 0.05 (^∗^). MRL, Murphy Roths Large; MSCs, mesenchymal stem cells; siRNA, small interfering RNA.

With regard to the increased expression of *Pycr1* during chondrogenesis, in particular for MRL MSCs, we next asked whether MSC chondrogenic differentiation could be regulated by a cell-autonomous function of *PYCR1*. To address that question, we used the siRNA approach to knock down the expression of *Pycr1* in MRL MSCs. Forty-eight hours post-transfection (at day 0 of MSC chondrogenesis) of MSCs with a siRNA against *Pycr1* (siPycr1), *Pycr1* expression was reduced by 70% compared with the MSCs transfected with the control siRNA (siCTL) (Figure 2A). The silencing of *Pycr1* in MRL MSCs did not modify their proliferation rate (Figure 2B). Then, MSC chondrogenic differentiation was induced by culture in micropellet in the presence of TGFβ3 for 21 days. While we observed a significant increase of *Pycr1* during chondrogenesis in MSCs transfected with the siCTL, *Pycr1* expression level did not change within differentiating MSCs transfected with si*Pycr1* (Figure 2A). Moreover, we assessed whether the downregulation of *Pycr1* altered the chondrogenic differentiation of MSCs; and we found, at early stages, that si*Pycr1*-transfected MSCs formed flat micropellets with a reduced density as compared with the micropellets formed with MSCs transfected with the si*Ctl* (data not shown). Moreover, *Pycr1* knockdown significantly reduced the expression, *Col2B*, a mature chondrocyte marker, by day 21 (Figure 2C).

### Pycr1 Is Necessary for Murphy Roths Large Mesenchymal Stem Cell Glycolytic Metabolism

**FIGURE 3:**
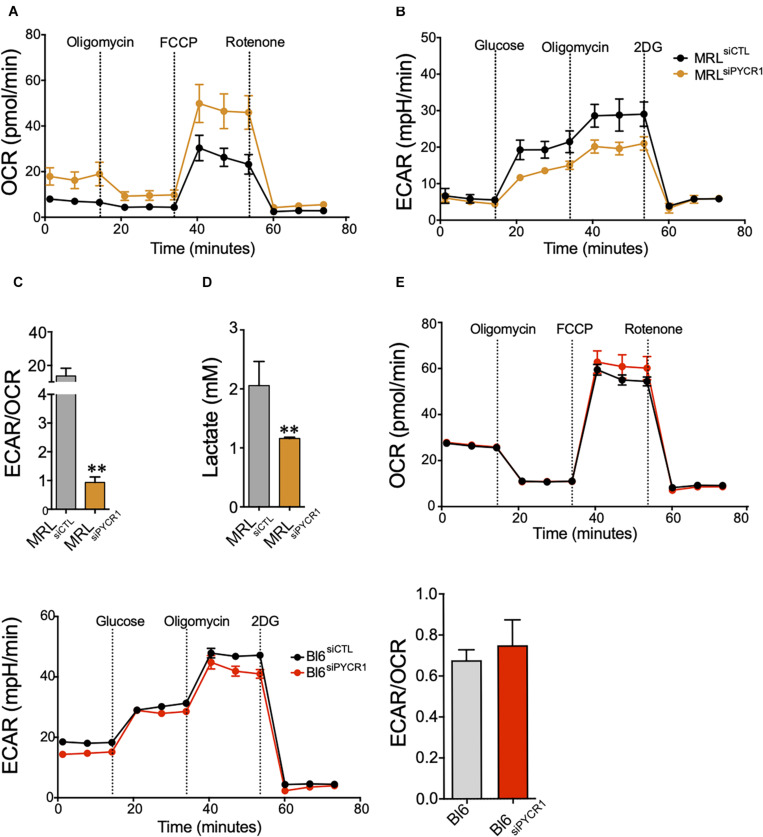
*Pycr1* induces a glycolytic metabolism on MRL MSCs. **(A,B)** The metabolic activity of MRL MSC siCTL (MRL MSCs transfected with the siCTL siRNA) control (black line) and siPYCR1 (orange line) was evaluated by measuring the OCR **(A)** or the ECAR **(B)** with Seahorse analyzer. The knockdown of *Pycr1* affects both the OCR and ECAR profiles, which translates to a preferentially oxidative metabolism. **(C)** The ratio between glycolytic rate and basal OCR confirms a significant decline in the glycolytic metabolism of MRL siPYCR1. **(D)** Similarly, we observed a significant decrease in lactate concentration in the extracellular media, as a result of the diminished glycolytic activity on MRL siPYCR1, compared with MRL siCTL. Results represent the mean ± SEM of three independent experiments with five different replicates each time. Statistics: Mann–Whitney unpaired *t*-test. ^∗∗^: *p* < 0.01. **(E)** The metabolic activity of BL6 MSC siCTL and BL6 MSC siPYCR1 was evaluated by measuring the OCR, ECAR and the ratio ECAR/OCR with Seahorse analyzer. MRL, Murphy Roths Large; MSCs, mesenchymal stem cell; siRNA, small interfering RNA.

PYCR1 activity has been linked to the glycolytic pathway through the production of NAD^+^ (Liu et al., 2015). Therefore, we wondered whether the high expression of *Pycr1* in MRL MSCs could regulate MRL MSC metabolism. We quantified, in both BL6 MSCs and MRL MSC, the OCR (Figure 3A) and their ECAR (Figure 3B), which are associated with OXPHOS and glycolysis, respectively. Overall, MRL MSCs showed an active glycolysis, which was partially controlled by *Pycr1* expression (Figures 3A,B). Indeed, the knockdown of *Pycr1* in MRL MSCs induces a switch from glycolysis to OXPHOS as revealed by the ratio of ECAR to OCR, which was also significantly lower in MRL MSCs deficient for *Pycr1* than in MSCs transfected with siCTL (Figure 3C). Then, we evaluated the lactate production (Figure 3D). In contrast, the knockdown of *Pycr1* in BL6 MSCs did not modify their metabolism (Figure 3E). Our results showed *Pycr1* downregulation induces a reprogramming of MRL MSC metabolism specifically associated with a decreased lactate concentration in the extracellular media of MRL MSCs deficient for *Pycr1* (Figure 3D). Altogether, these results provide evidence for the role of *Pycr1* in the cytosolic glycolytic activity in MRL MSCs.

### Pycr1 Is Necessary for the Maintenance of Chondrocyte Phenotype

**FIGURE 4:**
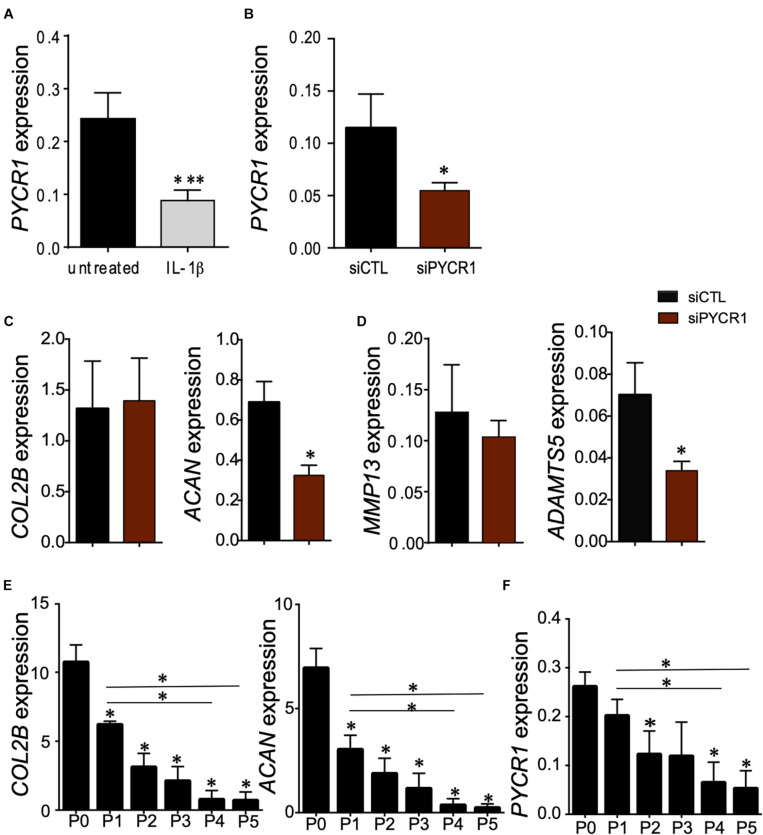
*Pycr1* silencing, a novel method to generate mouse IL-1β-treated chondrocytes. **(A)** Scheme showing the generation of chondrocytes with OA features with IL-1β treatment of freshly isolated chondrocytes. RT-qPCR analysis of *Pycr1* expression level in untreated healthy chondrocytes (untreated) and in IL-1β-treated chondrocytes (IL-1β). **(B)** RT-qPCR analysis of *Pycr1* expression level in chondrocytes transfected with a siRNA against PCYR1 (siPYCR1). **(C)** RT-qPCR analysis of the chondrocyte anabolic markers *Col2B* and *Acan* and **(D)** chondrocyte catabolic markers *Mmp13* and *Adamts5* in chondrocytes transfected with a control siRNA (siCTL) or a siRNA against *Pycr1* (siPYCR1). **(E)** RT-qPCR analysis of the chondrocyte anabolic markers *Col2B* and *Acan* and **(F)** *Pycr1* in mouse chondrocytes induced differently after serial passages in culture [from passage 0 (P0) to passage 5 (P5)]. Results represent the mean ± SEM of three independent experiments. Statistics: Mann–Whitney test, two-tailed. When not indicated P0 versus P1, P2, P3, P4, and P5. *p*-values < 0.05 (^∗^). OA, osteoarthritis; siRNA, small interfering RNA.

Articular chondrocytes are exposed to low O_2_ microenvironment *in vivo* and generate ATP by glycolysis (Lane et al., 2015). Under physiological conditions, chondrocytes rely on glycolysis to meet the cellular energy requirements, but when challenged with a stress or in OA, chondrocytes modify their mitochondrial respiration. This metabolic flexibility of chondrocytes is critical for their survival when stressed (Lane et al., 2015). We thus wondered whether the expression level of *Pycr1*, critical for cell glycolytic metabolism and function, could be modulated in chondrocytes exposed to different stresses. First, we assessed the modulation of *Pycr1* expression level in an *in vitro* model of chondrocyte inflammation that consists in treating freshly isolated chondrocytes with IL-1β (Ruiz et al., 2020). IL-1β treatment induced a chondrocyte model that reproduces the main OA chondrocyte features, namely, decreased expression of anabolic markers and increased expression of catabolic and inflammatory markers. In this model, IL-1β-treated chondrocytes exhibit a reduced expression of *Col2B* and *Acan* and increased expression of *Mmp13* and *Adamts5* (Ruiz et al., 2020). We found that the treatment of chondrocytes with IL-1β induced a significant downregulation of *Pycr1* (Figure 4A) similar to the one observed after the transfection of the chondrocytes with si*Pycr1* (Figure 4B). *Pycr1* silencing in articular chondrocytes using the si*Pycr1* resulted in a downregulation of the chondrocyte anabolic marker *Acan* (Figure 4C). No effect was observed on chondrocyte catabolic markers *Mmp13* and *Adamts5* (Figure 4D). This result suggests that *Pycr1* is essential to maintain chondrocyte phenotype. To confirm a correlation between the decreased expression level of *Pycr1* and the loss of chondrocyte phenotype, we then used the model of dedifferentiated chondrocytes (Monteagudo et al., 2017). In this model, chondrocytes progressively dedifferentiate upon serial passages in culture as revealed by the reduced expression level of chondrocyte anabolic markers (Monteagudo et al., 2017). Here, we found that in parallel to the progressive downregulation of *Col2B* and *Acan* in chondrocytes undergoing serial passages (Figure 4E), *Pycr1* was also progressively lost (Figure 4F).

Altogether, these results reveal that *Pycr1* loss parallels the loss of chondrocyte anabolic markers that characterize chondrocytes with OA features.

### Pycr1 Is Necessary for the Chondroprotective Potential of Murphy Roths Large Mesenchymal Stem Cell on the *in vitro* Model of Chondrocyte Inflammation

**FIGURE 5:**
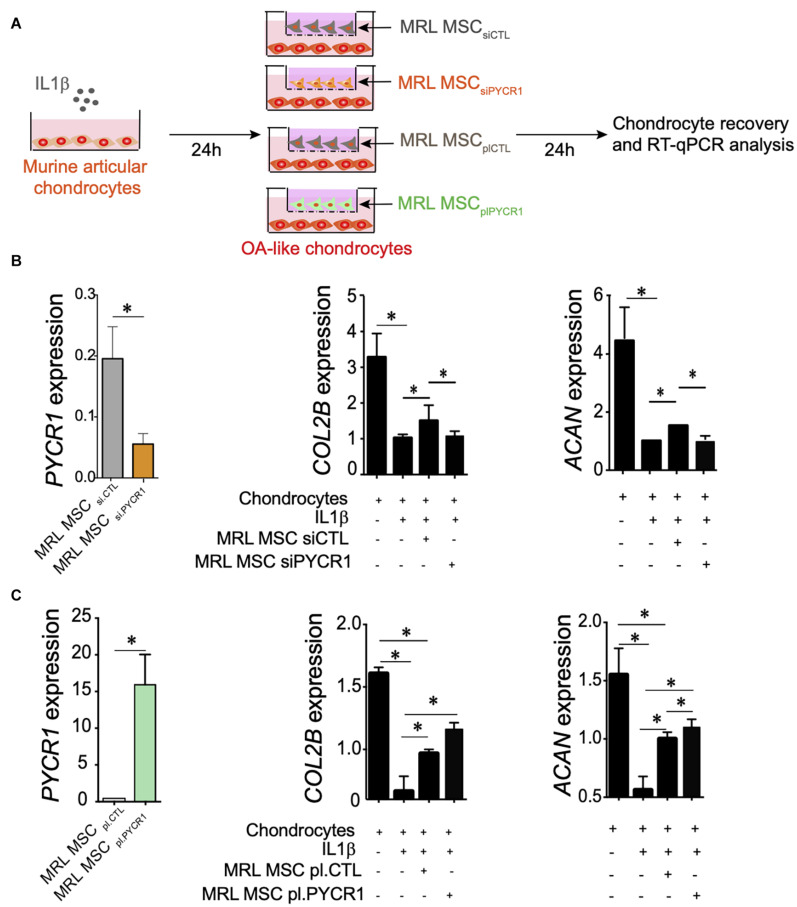
*Pycr1* regulates the chondroprotective abilities of MRL MSCs. **(A)** Scheme illustrating the different coculture conditions using chondrocytes with OA features induced by a 24-h incubation of freshly isolated chondrocytes with IL-1β and MRL MSCs. MRL MSCs were either transfected with a control siRNA (MRL MSC_siCTL_), a siRNA against *Pycr1* (MRL MSC_siPYCR1_), an empty vector control (MRL MSC_plCTL_), or a plasmid encoding *Pycr1* (MRLMSC_plPYCR1_). IL-1β-treated chondrocytes and MRL MSCs were cultured during 24 h before to be collected for RT-qPCR analysis. **(B)** RT-qPCR analysis of different chondrocyte markers, *Col2B* and *Acan*, in healthy chondrocytes and IL-1β-treated chondrocytes cultured alone or with either MRL MSC_siCTL_ or MRL MSC_siPYCR1_. **(C)** RT-qPCR analysis of different chondrocyte markers, *Col2B* and *Acan*, in healthy chondrocytes and IL-1β-treated chondrocytes cultured alone or with either MRL MSC_plCTL_ or MRL MSC_plPYCR1_. Results represent the mean ± SEM obtained with three biological replicates. Statistics: Mann–Whitney test, two-tailed. *p*-Values < 0.05 (^∗^). MRL, Murphy Roths Large; MSC, mesenchymal stem cell; OA, osteoarthritis.

We then asked whether *PYCR1* could be required for the chondroprotective properties of MSCs (Maumus et al., 2013b; Ruiz et al., 2020). To that end, we tested the effect of *Pycr1* silencing on MRL MSC chondroprotective effects on the IL-1β-induced chondrocyte model. While coculture of IL-1β-treated chondrocytes with MRL MSCs transfected with a si*Ctl* (MRL MSC_siCTL_) significantly upregulated the expression of chondrocyte anabolic markers including *Col2B* and *Acan*, MRL MSCs silenced for *Pycr1* (MRL MSC_siPYCR__1_) did not (Figures 5A,B). Conversely, we asked whether *Pycr1* overexpression on MRL MSCs would further enhance their chondroprotective properties on IL-1β-treated chondrocytes. To that end, MRL MSCs were transfected with either a plasmid encoding *Pycr1* (MRL MSC_plPYCR__1_) or an empty vector control (MRL MSC_plCTL_) prior to being cocultured with OA-like chondrocytes (Figure 5A). *Pycr1* was expressed 80-fold more in MRL MSC_plPYCR__1_ than in MRL MSC_plCTL_ (Figure 5C). Coculture of IL-1β-treated chondrocytes with MRL MSCs overexpressing *Pycr1* significantly upregulated the expression of chondrocyte anabolic markers *Col2B* and *Acan* (Figure 5C) as compared with the cocultures with MRL MSC_plCTL_. Altogether, these results indicate that *Pycr1* contributes to MRL MSC pro-anabolic function on chondrocytes.

### Pycr1 Is Necessary for the Regenerative Potential of Murphy Roths Large Mesenchymal Stem Cell in an Ear Punch Model

**FIGURE 6:**
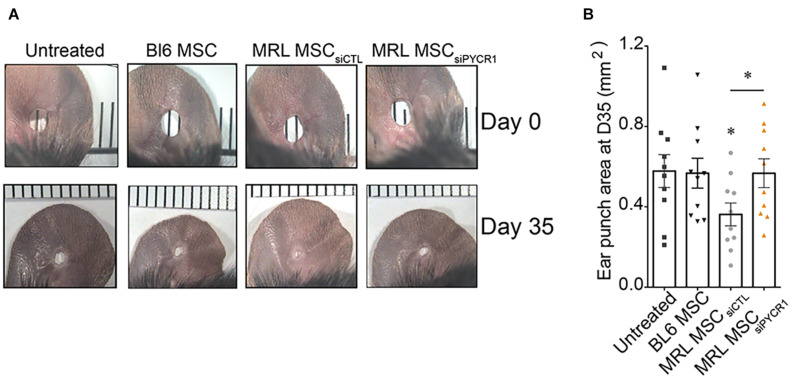
MRL MSCs induce tissue regeneration in BL6 mice in a *Pycr1*-dependent manner. **(A)** Pictures of the ear holes at day 0 and day 35 after wounding. The punch holes in the ears of BL6 mice were either untreated (untreated) or treated with MSCs. BL6 MSCs and MRL MSCs transfected with either siCTL (MRL MSC_siCTL_) or siPYCR1 (MRL MSC_siPYCR1_) were injected at the wound edges. **(B)** Quantification of the ear punch hole closure at day 35 (D35) using the ImageJ program to define the ear punch area. Results represent the mean ± SEM. *N*_*mice*_ = 10 per condition, Mann–Whitney test, two-tailed, when not indicated untreated versus MRL MSC_siCTL_, *p*-values < 0.05 (^∗^). MRL, Murphy Roths Large; MSC, mesenchymal stem cell.

The MRL mouse has been well described for its remarkable capacity for cartilaginous wound closure and regeneration (Clark et al., 1998). Two−millimeter punch wounds made into MRL/MpJ mice ears closed with regeneration after 30 days, whereas they did not close in the C57BL/6 mice. Histological analysis revealed a normal angiogenesis and chondrogenesis of the ear in contrast to control BL6 mice, which have unclosed ear holes (Clark et al., 1998). Going further, other studies have shown that the ear holes regenerate by the formation of a blastema−like structure, a highly proliferative structure composed of progenitor cells, leading to a scar-free vascularized tissue made of collagen, hair follicles, sebaceous glands, and even cartilage (Gawriluk et al., 2016). Since MRL MSCs have enhanced chondrogenic and chondroprotective properties as compared with BL6 MSCs, we investigated, *in vivo*, whether the regenerative potential of MRL mice was due to the intrinsic regenerative properties of their MSCs. To that end, non-regenerating BL6 mice were subjected to a through-and-through hole generated in the ear pinna using a 2-mm biopsy punch. Then, the wounded mice were either untreated or treated with MSCs injected along the wound edge. Measurements of the ear punch wound area at day 35 revealed that MRL MSCs induced a regenerative process leading to a significant decrease of the wound size, 37% or 36% reduction in area, as compared with the untreated mice (PBS injected) or mice treated with BL6 MSCs, respectively (Figures 6A,B). Then, we assessed, whether this regenerative process mediated by MRL MSCs was associated with their high expression level of *Pycr1*. The ear punch wound area of BL6 mice treated with MRL MSCs deficient for *Pycr1* (MRL MSC si_PYCR__1_) did not show any difference with the untreated or BL6 MSCs treated mice (Figures 6A,B). Overall, this finding suggests that the *in vivo* regenerative potential of MRL MSCs that we showed in BL6 mice depends on *Pycr1* expression level.

## Discussion

This study provides the first evidence that MRL MSCs exhibit enhanced chondrogenic, chondroprotective, and regenerative properties as compared with BL6 MSCs in a *Pycr1*-dependent manner.

We found that MRL MSCs induce toward the chondrogenic lineage differentiate faster and better than BL6 MSCs as revealed by the high expression level of chondrocyte markers as soon as day 14 of the MSC differentiation process that takes normally 21 days for MSCs. Herein, we evidenced a progressive increase in the expression level of *Pycr1* during the course of chondrogenic differentiation of MSCs and that the loss of *Pycr1* in MRL MSCs abolishes their chondrogenic potential as revealed by the decreased expression levels of chondrogenic markers that we studied at the mRNA expression level since we have previously shown a nice correlation between mRNA and protein of chondrogenic markers (Bouffi et al., 2010). This latter effect might be due, in part, to the fact that PYCR1 is the final enzyme in the biosynthesis of proline that makes up approximately 15% of collagen accounting for about two-thirds of articular cartilage dry weight. Proline, with also the post-translational modifications, are necessary for appropriate collagen synthesis, folding, and secretion (Guzy and Redente, 2021; Stum et al., 2021). Thus, further research is needed to determine the effect of *Pycr1* downregulation on the capacity of MSCs undergoing chondrogenesis to produce reduced level of PYCR1 and collagen. Of note, since a similar regulation between mRNA and protein levels of PYCR1 has been previously described (Huang et al., 2018; Weijin et al., 2019; Xiao et al., 2020), we mainly relied on the mRNA expression level of *Pycr1* to confirm *Pycr1* silencing and overexpression in the present study. Moreover, *PYCR1* mutations are deleterious for mitochondrial function and responsible for progeroid changes in connective tissues (Reversade et al., 2009). Thus, *Pycr1* silencing in MRL MSCs might have antagonized the intrinsic properties of MRL mouse cells that have retained some features of embryonic cells including their metabolism and the expression of stem cell markers such as *Nanog*, *Islet-1*, and *Sox2* (Naviaux et al., 2009). The retention of such embryonic features in adulthood is rare in mammals and might confer to MRL MSCs their enhanced differentiation potential in a *Pycr1*-dependent manner.

Moreover, PYCR1 participates to the upregulation of glycolysis through proline biosynthesis (Liu et al., 2015). We therefore studied the metabolism of MSCs derived from the superhealer MRL mice (MRL MSCs) and found a high glycolytic metabolism in those cells. Moreover, in loss-of-function experiments, we demonstrated that the glycolytic status of MRL MSCs is associated with a high expression level of *Pycr1*. Indeed, *Pycr1* silencing significantly reduces the ratios of ECAR to OCR that indicates a preference for OXPHOS over glycolysis in MRL MSCs deficient for *Pycr1*. Moreover, the lactate production by MRL MSCs silenced for *Pycr1* was also significantly reduced. This is in accordance with the capacity of PYCR1 to increase glycolysis (Liu et al., 2015) and the reduced O_2_ consumption and OXPHOS in MSCs during chondrogenesis, indicating a shift toward increased glycolysis (for review, see Shyh-Chang et al., 2013; Mobasheri et al., 2017; Zheng et al., 2021). During chondrogenesis, a rapid and significative reduction in oxygen consumption has been reported in MSCs not due to the chondrogenic differentiation *per se* but rather to the 3D pellet culture conditions. The expression levels of genes associated with glycolysis increased in MSCs that adopt a glycolytic metabolism and differentiate into chondrocytes (Pattappa et al., 2011). Moreover, under hypoxia that induces a high glycolytic metabolism, chondrogenesis is enhanced (Lennon et al., 2001; Wang et al., 2005; Xu et al., 2007; Markway et al., 2010). Altogether, these results suggest that MRL MSCs are more prone to differentiate into chondrocytes presumably due to their PYCR1-dependent glycolytic status.

Mature chondrocytes are highly glycolytic with a minimal oxygen consumption (Rajpurohit et al., 1996; Heywood and Lee, 2008). Downregulation of the chondrocyte markers such as *Col2B* and *Acan* in chondrocytes undergoing serial passages is correlated with *Pycr1* expression progressive loss. This was confirmed using another model of chondrocyte inflammation that induces their osteoarthritic-like dedifferentiation characterized by a loss of chondrocyte marker expression (Benya et al., 1978; Monteagudo et al., 2017), increasing their oxygen consumption and their OXPHOS metabolism (Heywood and Lee, 2008). Indeed, we demonstrated that in parallel to the progressive acquisition of an OA-like phenotype upon IL-1β exposure, chondrocytes exhibit a reduced expression of *Pycr1*. Going further, we showed that *Pycr1* silencing led to a significant decrease of chondrocyte markers confirming that *Pycr1* expression in articular chondrocytes is required for a functional phenotype. Altogether, our results show that PCYR1, pivotal for cell glycolytic metabolism, is required for healthy and functional articular chondrocytes. These results are in line with the effect induced by the treatment of chondrocytes with 2-deoxyglucose (2-DG), a chemical inhibitor of glycolysis, that highly reduces *Col2B* expression levels supporting the pivotal role of glycolytic energy production for cartilage matrix synthesis (Pfander et al., 2003, 2006).

Knockdown of *Pycr1* in MSCs also altered MSC chondroprotective properties on IL-1β-treated chondrocytes. We showed that while MRL MSCs protect IL-1β-treated chondrocytes from a loss of anabolic markers (*Acan* and *Col2B*), MRL MSCs silenced *Pycr1* did not. Moreover, we showed that *Pycr1* enhances the chondroprotective potential of MSCs as revealed by higher expression levels of anabolic markers in IL-1β-treated chondrocytes cocultured with MSCs overexpressing *Pycr1* as compared with IL-1β-treated chondrocyte coculture with control MSCs. Thus, we evidenced that the chondroprotective effect of MRL MSCs relies on *Pycr1* expression. The cytoprotective effect of MSCs has been associated with their glycolytic phenotype. Enhancement of glycolysis promotes MSC survival (Yang et al., 2019). The glycolytic metabolic state maintains MSC homeostasis by limiting reactive oxygen species (ROS) production through the cytoprotective effect of high glycolytic flux that enhances generation of antioxidant precursors (Galluzzi et al., 2013; Yuan et al., 2019). Our results suggest that *Pycr1* is pivotal for the chondroprotective high glycolytic flux mediated by MRL MSC.

The activation of glycolysis has been shown to play a pivotal role in regeneration (Magadum and Engel, 2018). The regenerative abilities of MRL mice have been associated with an increased glycolysis and a reduced OXPHOS (Naviaux et al., 2009). Moreover, during planarian tissue regeneration, the activation of glycolysis has been reported (Osuma et al., 2018). Glycolysis inhibition repressed regeneration of mouse neonatal hearts (Wang et al., 2018) and adult skeletal muscle (Fu et al., 2015). However, although regeneration is associated with glycolysis, the underlying mechanisms are poorly understood. Here, we show that MRL MSCs have enhanced regenerative properties and that their injection at the wound edges stimulates the regenerative process in non-regenerating BL6 mice. This regenerative potential exhibited by MRL MSCs required the expression of *Pycr1*, previously described to play a critical role in energy metabolism.

In conclusion, our findings demonstrate that the enhanced regenerative potential of MRL mice is attributed, in part, to their MSCs that exhibit PYCR1-dependent higher glycolytic potential, differentiation capacities, chondroprotective abilities, and regenerative properties than BL6 MSCs.

## Data Availability Statement

The original contributions presented in the study are included in the article/supplementary material, further inquiries can be directed to the corresponding author/s.

## Ethics Statement

The animal study was reviewed and approved by Ethical Committee for animal experimentation of the Languedoc-Roussillon before to initiate the study (approval CEEA−LR−12117).

## Author Contributions

FD designed the all project and the experiments. DN, FDC, PP, PL-C, and CJ contributed to design and interpreted the data for the work. GT, RC-L, AB, PL-C, and MR performed the experiments and analyzed the results. FD wrote the manuscript with the input of PL-C and CJ. All authors revised and gave final approval of the manuscript.

## Conflict of Interest

FDC and PP were employed by the Institut de Recherches Servier. The authors declare that this study received funding from Servier. The funder was not involved in the study design, collection, analysis, interpretation of data and the writing of this article for publication.
